# Compressed Sensing: Applications in Radar and Communications

**DOI:** 10.1155/2016/5407415

**Published:** 2016-03-30

**Authors:** Sandra Costanzo, Álvaro Rocha, Marco Donald Migliore

**Affiliations:** ^1^Dipartimento di Ingegneria Informatica, Modellistica, Elettronica e Sistemistica, Università della Calabria, 87036 Rende, Italy; ^2^Departamento de Engenharia Informática, Universidade de Coimbra, Coimbra, Portugal; ^3^Università degli Studi di Cassino e del Lazio Meridionale, Cassino, Italy

*If information bandwidth less than total bandwidth, then should be able to sample below Nyquist without information loss and recover missing samples by convex optimization. (Emmanuel Candès, “Compressive Sensing—A 25 Minute Tour,” EU-US Frontiers of Engineering Symposium, Cambridge, September 2010)*

Compressed Sensing is an emerging approach exploiting the sparsity feature of a signal to give accurate waveform representation at reduced sampling rate, below the Shannon-Nyquist conditions, thus leading to efficient radar and communication systems, with reduced complexity and cost.

The aim of this special issue is to provide an international forum for experts and researchers working in the area of Compressed Sensing applied to radar and communication contexts, in order to explore the state of the art of such techniques and to present new advanced concepts and results.

This special issue collects 6 papers from 14 authors belonging to different countries and institutions. It summarizes the most recent developments and ideas on emerging Compressed Sensing approaches, with particular focus addressed to the following issues:

- Compressed Sensing for signal processing.
- Compressed Sensing for MIMO architectures.
- Compressed Sensing for inverse scattering.
- Compressed Sensing for high-resolution radars.
- Compressed Sensing for wireless communications and networks.

In the paper by S. Costanzo entitled “Compressed Sensing/Sparse-Recovery Approach for Improved Range Resolution in Narrow-Band Radar,” a Compressed Sensing formulation is adopted to enhance the range resolution of narrow-band radars.

In the paper by M. Minner entitled “Compressed Sensing in On-Grid MIMO Radar,” the feasibility of Compressed Sensing-based MIMO radar to detect on-grid targets in the azimuth, time-delay, and Doppler domain is investigated.

The paper by M. E. Domínguez-Jiménez et al. entitled “Estimation of Symmetric Channels for Discrete Cosine Transform Type-I Multicarrier Systems: A Compressed Sensing Approach” explores the application of Compressive Sensing to the problem of channel estimation for multicarrier communications.

In the paper by S. K. Bolisetti et al. entitled “Subspace Compressive GLRT Detector for MIMO Radar in the Presence of Clutter,” the target detection performances of MIMO radars in the presence of clutter are optimized by adopting a compressive GLRT detector.

The paper by M. T. Bevacqua and L. Di Donato entitled “Improved TV-CS Approaches for Inverse Scattering Problem” jointly exploits the Compressed Sensing and Total Variation approaches to improve the solution of inverse scattering problems arising in radar applications.

Finally, in the paper by A. Budillon and G. Schirinzi entitled “Support Detection for SAR Tomographic Reconstructions from Compressive Measurements,” the problem of detecting multiple scatterers in the same range azimuth resolution cell from a compressive number of multibaseline SAR images is dealt with.

*Sandra Costanzo* *Sandra Costanzo* *Álvaro Rocha* *Álvaro Rocha* *Marco Donald Migliore* *Marco Donald Migliore*

